# Electromagnetic Treatment to Old Alzheimer's Mice Reverses β-Amyloid Deposition, Modifies Cerebral Blood Flow, and Provides Selected Cognitive Benefit

**DOI:** 10.1371/journal.pone.0035751

**Published:** 2012-04-25

**Authors:** Gary W. Arendash, Takashi Mori, Maggie Dorsey, Rich Gonzalez, Naoki Tajiri, Cesar Borlongan

**Affiliations:** 1 Department of Cell Biology, Microbiology, and Molecular Biology, University of South Florida, Tampa, Florida, United States of America; 2 The Florida Alzheimer's Disease Research Center, Tampa, Florida, United States of America; 3 Departments of Biomedical Sciences and Pathology, Saitama Medical Center and Saitama Medical University, Kawagoe, Saitama, Japan; 4 The University of South Florid Health Byrd Alzheimer's Institute, Tampa, Florida, United States of America; 5 SAI of Florida, Redington Beach, Florida, United States of America; 6 Center of Excellence for Aging and Brain Repair, Department of Neurosurgery and Brain Repair, University of South Florida, Morsani College of Medicine, Tampa, Florida, United States of America; Alexander Flemming Biomedical Sciences Research Center, Greece

## Abstract

Few studies have investigated physiologic and cognitive effects of “long-term" electromagnetic field (EMF) exposure in humans or animals. Our recent studies have provided initial insight into the long-term impact of adulthood EMF exposure (GSM, pulsed/modulated, 918 MHz, 0.25–1.05 W/kg) by showing 6+ months of daily EMF treatment protects against or reverses cognitive impairment in Alzheimer's transgenic (Tg) mice, while even having cognitive benefit to normal mice. Mechanistically, EMF-induced cognitive benefits involve suppression of brain β-amyloid (Aβ) aggregation/deposition in Tg mice and brain mitochondrial enhancement in both Tg and normal mice. The present study extends this work by showing that daily EMF treatment given to very old (21–27 month) Tg mice over a 2-month period reverses their very advanced brain Aβ aggregation/deposition. These very old Tg mice and their normal littermates together showed an increase in general memory function in the Y-maze task, although not in more complex tasks. Measurement of both body and brain temperature at intervals during the 2-month EMF treatment, as well as in a separate group of Tg mice during a 12-day treatment period, revealed no appreciable increases in brain temperature (and no/slight increases in body temperature) during EMF “ON" periods. Thus, the neuropathologic/cognitive benefits of EMF treatment occur without brain hyperthermia. Finally, regional cerebral blood flow in cerebral cortex was determined to be reduced in both Tg and normal mice after 2 months of EMF treatment, most probably through cerebrovascular constriction induced by freed/disaggregated Aβ (Tg mice) and slight body hyperthermia during “ON" periods. These results demonstrate that long-term EMF treatment can provide general cognitive benefit to very old Alzheimer's Tg mice and normal mice, as well as reversal of advanced Aβ neuropathology in Tg mice without brain heating. Results further underscore the potential for EMF treatment against AD.

## Introduction

Despite the best efforts of pharmaceutical industry and academia, no new drugs against Alzheimer's Disease (AD) have been developed since 2003 [1]. Moreover, currently available drugs (acetylcholinesterase inhibitors and/or N-metyle D-aspartate (NMDA) antagonists) only treat/mask AD symptoms for about one year, if at all - none of them directly slow or lessen AD pathogenesis itself. In view of the universal failure of every major drug trial to alter the course of AD, it is time to think outside the “pharmaceutical box" by considering non-pharmaceutical approaches that are safe, disease modifying, and can be expeditiously explored to treat AD. We propose high frequency electromagnetic field (EMF) treatment could be that approach, based on several epidemiologic studies [2], [3] and our recently completed EMF studies in Alzheimer's transgenic (Tg) mice [4], [5].

In humans, high frequency EMF exposure/treatment studies have essentially involved “cell phone level" EMF parameters (pulsed, modulated and primarily GSM), in large part because of initial concerns that high frequency EMF exposure may induce health problems such as brain cancer [6], [7]. However, the recent 13-nation INTERPHONE study [8], as well as analytic findings from NIEHS [9] and numerous epidemiologic studies [10]–[12], all collectively conclude that there is no consistent evidence that long-term exposure of adults or children/adolescents to cell phone level EMFs causes brain tumors, or very likely any other health problems for that matter. In concert with these studies alleviating safety issues related to high frequency EMF exposure, dozens of studies have investigated potential cognitive and physiologic (i.e., EEG, cerebral blood flow, and auditory processing) effects of cell phone level EMF exposure. With rare exception [13], [14], these studies only involved brief (3–120 minute), single EMF exposure at GMS, CW, or UMTS cell phone parameters given to normal subjects. Not surprisingly, recent reviews/meta-analyses find these “acute" exposure studies to result in no significant beneficial or impairing effects on cognitive performance [15], [16]. Nonetheless, several PET studies have reported that acute, single-exposure EMF treatment can affect regional cerebral blood flow [17], [18] and increase brain glucose utilization [19], thus suggesting that even acute high frequency EMF treatment can affect brain neuronal activity.

Although results from acute, single EMF exposure studies are insightful, they are most probably not indicative of the physiologic and cognitive effects of long-term/daily EMF exposure (i.e. the EMF exposure typical of cell phone users or the repeated EMF treatments almost certainly required for any clinical EMF applications). In this context, no controlled human studies have investigated the “long-term" effects of high frequency EMF treatment in normal or AD subjects over weeks, months, or years. Nonetheless, two epidemiologic studies have provided initial human evidence that years of high frequency EMF exposure are associated with cognitive benefit. One of these studies found that heavy cell phone use over several years resulted in better performance of normal subjects on a word interference test [2], while the other study reported that long-term cell phone users (>10 years) had a 30–40% decreased risk of hospitalization due to AD and vascular dementia [3].

The lack of controlled human studies investigating cognitive effects of “long-term" EMF exposure/treatment has at least been partially negated by our highly controlled EMF treatment studies in AD Tg mice and littermate non-transgenic (NT) mice [4], [5]. In the first long-term, high frequency EMF treatment study evaluating cognition in adult humans or animals [4], we reported that treatment (at cell phone levels of 918 MHz/0.25–1.05 W/kg; pulsed and modulated) over 7–9 months prevented or reversed cognitive impairment in AD Tg mice bearing the APPsw mutation. Even normal mice showed EMF-induced cognitive enhancement in that initial study. For AD mice, the primary mechanism of cognitive benefit appears to be a suppression of brain Aβ aggregation into neuritic plaques, presumably resulting in greater Aβ efflux from the brain [4]. Moreover, the cognitive benefits of long-term EMF treatment to both AD mice and normal mice occurs without any evidence of tissue abnormalities in either the brain or peripheral tissues, without any evidence of increased oxidative stress in the brain, and without any increase in DNA damage to circulating blood cells. Thus, long-term EMF treatment in mice appears safe in having no deleterious side effects across multiple sensitive markers of brain/body function.

In a second study that involved AD Tg mice bearing the APPsw+PS1 double mutation, we reported that daily EMF treatment for one month enhances the impaired brain mitochondrial function of these AD mice, as well as the brain mitochondrial function of normal mice [5]. These EMF-induced mitochondrial enhancements occurred through “non-thermal" mechanisms because brain temperatures were either stable or decreased during and after daily high frequency EMF treatments. Since this EMF-induced mitochondrial enhancement in AD mice was linked to dramatic 5–10 fold elevations in soluble Aβ within the same mitochondria, EMF treatment disaggregated toxic Aβ oligomers therein, apparently resulting in very high monomeric Aβ levels (which are innocuous to mitochondrial function). Our mitochondrial function results in Dragicevic et al. [5] collectively suggest that brain mitochondrial enhancement may be a primary mechanism through which long-term EMF treatment provides cognitive benefit to both AD mice and NT mice.

In a third study, we have most recently reported that two months of daily EMF treatment enhances neuronal activity in the entorhinal cortex of aged Alzheimer's Tg mice and littermate NT mice [20]. This EMF-induced enhancement of neuronal activity was temporally linked to cognitive benefit in the same animals. Based on these results, we have suggested that EMF treatment could be a viable approach to counter the neuronal hypo-activity that occurs very early in AD pathogenesis [20].

It is noteworthy that our prior EMF studies [4], [5], [20] identified the first biologic mechanisms that could explain the EMF-induced cognitive benefits, which we also reported in normal and Alzheimer's Tg mice (i.e., anti-Aβ aggregation, mitochondrial enhancement, and enhanced neuronal activity). The fact that our long-term EMF treatment involves pulsed, modulated GSM signal is important because a recent, comprehensive review concluded that EMF-induction of biologic effects occurs primarily with GSM-type modulation and a pulsed signal - not continuous wave or UMTS fields [21].

Our initial behavioral study in AD Tg mice involved long-term EMF treatment to young adult APPsw mice (from 2–7.5 months of age), as well as to older APPsw adults (from 5–13.5 months of age) [4]. Inasmuch as Aβ pathology was not yet well established when treatment began for these mice, the beneficial effects reported were most relevant to human EMF treatment in pre-symptomatic/prodromal AD or in mild cognitive impairment (MCI), the prelude to AD. The present study extends our earlier findings by evaluating the impact of long-term EMF treatment given to very old 21–26 month-old APPsw and APPsw+PS1 mice, both of which bear much heavier brain Aβ burdens/Aβ levels than the APPsw mice in our initial work. In these aged mice with advanced Aβ pathology, we evaluated an array of behavioral, neuropathologic, and physiologic measures to get a clearer understanding of how long-term EMF treatment might impact the aged and heavily Aβ-burdened brain. We report a profound ability of long-term EMF treatment to reverse brain Aβ deposition, induce changes in regional cerebral blood flow, and provide selected cognitive benefits - all without induction of brain hyperthermia.

## Results

### Behavioral assessment during long-term EMF treatment

In Study I, behavioral testing of aged Tg and NT mice between 1 and 2 months into daily EMF treatment indicated no deleterious effects of EMF treatment on sensorimotor function (Table 1). For both Tg and NT mice, general activity/exploratory behavior was unaffected by EMF treatment, as indexed by open field activity and Y-maze choices made. As well, balance and agility abilities were not impacted in either Tg or NT mice by EMF treatment, as indexed by balance beam and string agility performance. In both of these tasks, however, an overall effect of genotype was presence, with Tg mice having poorer balance/agility compared to NT mice irrespective of EMF treatment (p<0.002). Finally, visual acuity testing in the visual cliff task at the end of behavioral testing (2 months into EMF treatment) indicated no deleterious effects of EMF treatment on vision in either Tg or NT mice.

**Table 1:** Sensorimotor measures in NT and Tg mice given long-term EMF treatment.

	NT	NT+EMF	Tg	Tg+EMF
**Open Field** (line crossings)	91±21	115±15	90±28	122±34
**Y-maze** (# of choices)	24±4	22±1	26±5	24±3
**Balance Beam** (sec)	4.2±0.2	4.6±0.7	2.5±0.6	1.5±0.8
**String Agility** (score)	3.0±0.6	2.6±0.4	1.5±0.5	0.5±0.3
**Visual Cliff** (score)	2.0±0	1.8±0.2	1.8±0.2	1.9±0.1

**Figure 1:**
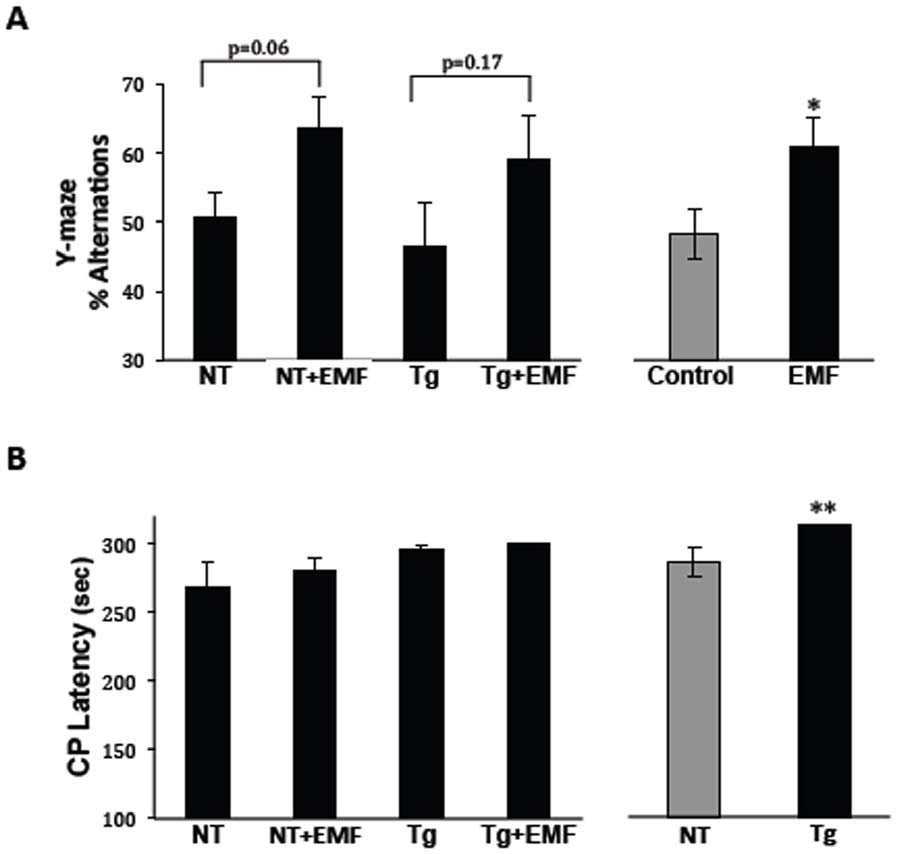
Cognitive performance of non-transgenic (NT) and APPsw transgenic (Tg) mice in the Y-maze task of spontaneous alternation (Fig. 1A) and the circular platform task of spatial/reference memory (Fig. 1B). (Fig. 1A) Both NT and Tg mice given EMF treatment exhibited nearly significant increases in Y-maze percent alternation. For both genotypes combined, a significant increased in percent alternation was evident in EMF-treated mice. *p<0.05 vs. control. (Fig. 1B) EMF treatment did not improve the poor performance of NT and Tg mice in the circular platform task, although Tg mice were impaired even more than NT mice irrespective of treatment. **p<0.02 vs. NT.

For cognitive-based tasks/measures, EMF effects were task specific with benefits observed in the Y-maze task, but no effects in either the circular platform or radial arm water maze (RAWM) tasks. In the Y-maze alternation task of general mnemonic function, both Tg and NT mice being given EMF treatment showed near-significance increases in percent alternation compared to their respective controls (Fig. 1A, left). Because there was no difference in performance of Tg and NT mice, these genotypic groups were combined to determine if an overall EMF treatment effect was present. Indeed, a significant increase in spontaneous alternation percentage was evident irrespective of genotype (Fig. 1A, right), indicating a beneficial effect of EMF treatment on general mnemonic function. In the circular platform task of spatial/reference memory, Tg mice were impaired vs. NT controls during the final (2^nd^ block) of testing, irrespective of whether they were receiving EMF treatment or not (Fig. 1B). Furthermore, EMF treatment did not improve the poor performance (e.g, high escape latencies) of both Tg and NT mice in this task.

**Figure 2:**
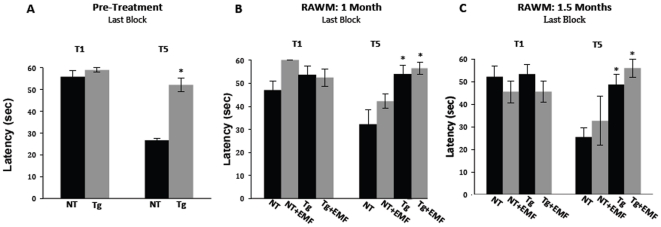
Working memory in the radial arm water maze (RAWM) task pre-treatment, 1 month, and 1.5 months into EMF treatment for the naïve first trial (T1) and working memory trial (T5) of APPsw transgenic (Tg) and non-transgenic (NT) mice. (Fig. 2A) Pre-treatment RAWM testing revealed Tg mice to be impaired vs. NT mice during working memory Trial 5 on the last block of testing. *p<0.002 vs. NT; (Fig. 2B) and (Fig. 2C). At both 1 month and 1.5 months into EMF treatment, Tg mice continued to be impaired in working memory (T5) performance on the last block of testing, irrespective of whether they had been receiving EMF treatment or not. *p<0.01 or higher level of significance vs. NT.

For the RAWM task of working memory, all animals were tested prior to the start of EMF treatment to establish baseline performance levels and to determine if a transgenic effect was present. Throughout pre-treatment RAWM testing, both Tg and NT mice showed the high escape latencies typically seen during the naïve first trial (T1), as exemplified by the last block of pre-treatment testing (Fig. 2A). By contrast, Tg mice showed a severe working memory impairment compared to NT mice at individual test blocks and overall, as exemplified by their substantially higher escape latencies during working memory Trial 5 (T5) for the last block of pre-treatment testing (Fig. 2A). Following completion of pre-treatment testing, Tg mice were divided into two sub-groups balanced in RAWM performance (as were NT mice), with one sub-group receiving EMF treatment and the other group not. Ensuing RAWM testing at both 1 month and 1.5 months into EMF treatment continued to show substantially impaired working memory (T5) performance in Tg mice vs. NT controls, irrespective of whether they were receiving EMF treatment or not (Figs. 2B, C). The similar T5 working memory impairment of Tg+EMF mice and Tg controls (evident during individual blocks and overall) is exemplified by the last block of testing, as shown in Figs. 2B and C.

Thus, EMF-induced cognitive benefits to very old AD and NT mice were selective in enhancing general mnemonic function (Y-maze alternation), but not impacting spatial reference learning/memory (circular platform) or working memory (radial arm water maze).

### Body/brain temperature recording during long-term EMF treatment

#### Study I

**Figure 3:**
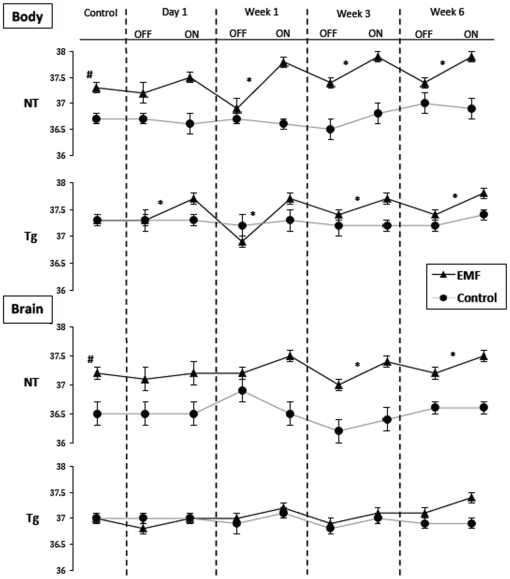
Body and brain temperature measurements for non-transgenic (NT) and APPsw transgenic (Tg) mice recorded prior to the start of EMF treatment (control), and at 1 Day, 1 week, 3 weeks, and 6 weeks into EMF treatment. Control NT and Tg mice (no EMF exposure) maintained stable body and brain temperatures throughout the 6 week recording period. By contrast, both NT and Tg mice being treated with EMF experienced small, but significant increases in body temperature during ON periods by 1 week into treatment and time points thereafter. Although brain temperature of EMF-treated Tg mice remained stable during ON periods through the 6 week recording period, EMF-treated NT exhibited small (but significant) increases in brain temperature during ON periods at 3 and 6 weeks into EMF treatment. *p<0.05 or higher level of significance for OFF vs. ON recordings (via paired t-test) at that time point. ^#^p<0.02 vs. NT control on that day.

Body and brain temperature measurements were attained from aged animals in Study I before start of EMF treatment (control) and at 1, 3, and 6 weeks into treatment (final temperature measurements were unfortunately not taken at the 2-month termination point of this study). Throughout the 6-week study period, body and brain temperature recordings indicated very stable temperature in control NT and control APPsw (Tg) mice not being given EMF treatment (Fig. 3). By contrast, body temperature for both EMF-treated NT and Tg mice was modestly elevated by 0.5–0.9°C during ON periods compared to OFF periods, from 1 week into EMF treatment onward through treatment. For Tg mice, this increase in body temperature during ON periods was evident even on the first day of EMF treatment. During EMF OFF periods for both NT and Tg mice, body temperature always came back down to their pre-treatment levels. Since body temperature before start of EMF treatment was identical for Tg mice (but not NT mice) to be given EMF or sham treatment, temperature comparisons between these two groups throughout the EMF treatment period also revealed that the elevated body temperatures of Tg mice during ON periods always came back down to sham control levels during OFF periods.

As indicated in Fig. 3, brain temperature in control NT and Tg mice was usually 0.3–0.4°C lower than body temperature, which is typically the case for rodents [22]. As with body temperatures, brain temperature measurements in control NT and Tg mice (not given EMF treatment) were very stable throughout the study. In EMF-treated NT mice, elevations of 0.3–0.4°C in brain temperature during ON periods were evident and significant by 3 weeks into treatment (Fig. 3). In EMF-treated Tg mice, however, only trends for a slight increase in brain temperature were present during ON periods. Thus, even with peripheral increases in temperature during ON periods, brain temperature remained stable or was only elevated minimally through 6 weeks of EMF exposure. Following any brain temperature elevations during ON periods, brain temperature always returned to pre-treatment levels during OFF periods.

#### Study II

**Figure 4:**
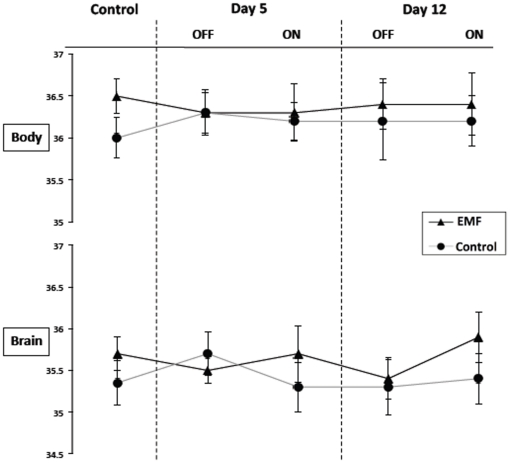
Body and brain temperature measurements for APPsw+PS1 transgenic (Tg) mice recorded prior to the start of EMF treatment (control), as well as at 5 days and 12 days into EMF treatment. For both control and treatment time points, there were no differences between EMF-treated and control Tg mice for either body or brain temperatures. No significant differences in OFF vs. ON temperatures (via paired t-test) were evident in EMF-treated Tg mice.

For the aged APPsw+PS1 (Tg) mice in Study II, body and brain temperature measurements were taken before the start of EMF treatment, as well as at 5 and 12 days into treatment (Fig. 4). Though still modest, the difference between body and brain temperature measurements for control APPsw+PS1 mice throughout this study was larger (0.7–0.9°C) than for the body/brain temperature differences of APPsw mice throughout Study I. Despite receiving the same daily EMF exposure as APPsw mice in Study I, APPsw+PS1 mice in this study showed no significant increase in body or brain temperature during ON periods at 5 and 12 days into EMF treatment. For all time points evaluated, there were no differences between EMF-treated and control Tg mice in either body or brain temperature.

### Cerebral blood flow measurements during long-term and sub-chronic EMF treatment

**Figure 5:**
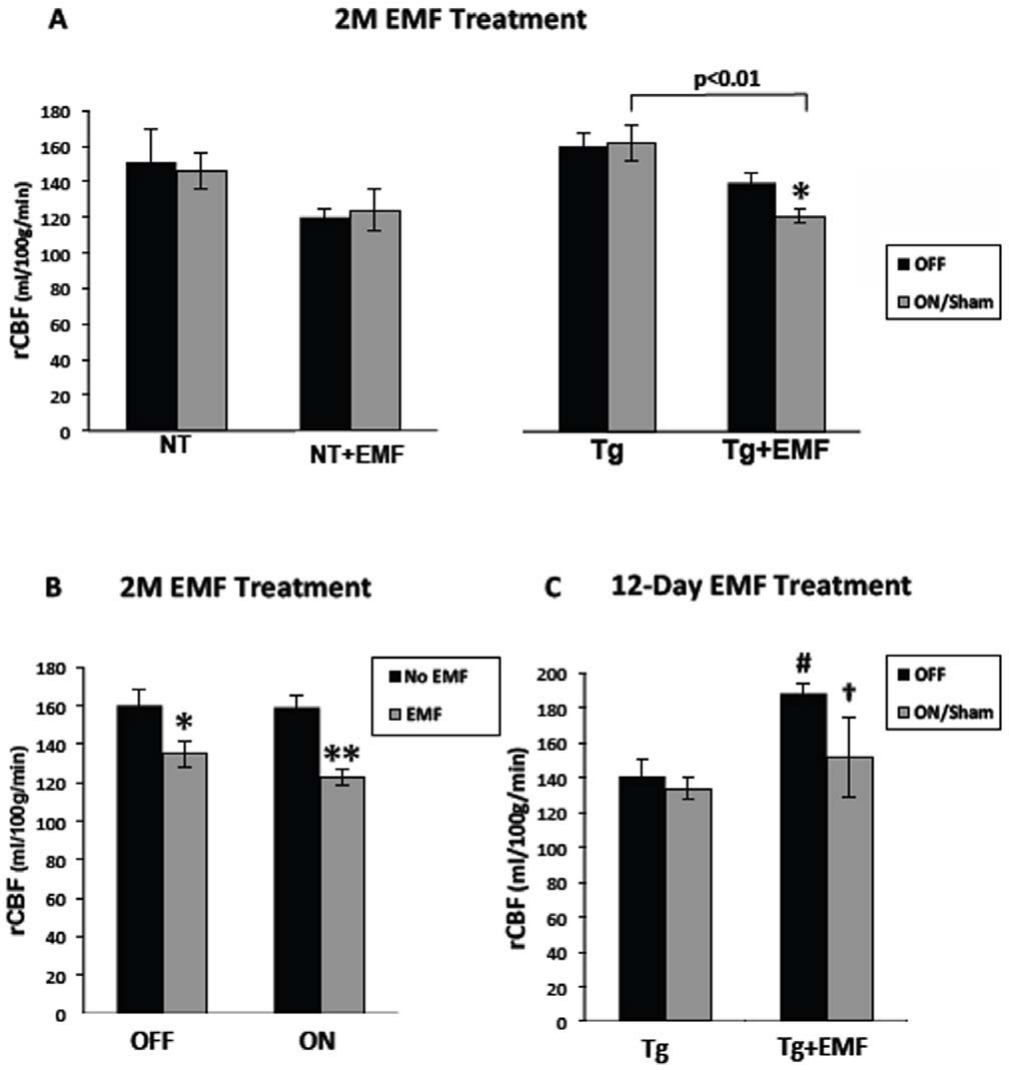
Regional cerebral blood flow (rCBF) in cerebral cortex of NT and Tg mice in Studies I and II obtained by Laser Doppler measurements at the end of their 2 month and 12-day EMF treatment periods, respectively. (Fig. 5A) At 2 months into EMF treatment for Study I, APPsw transgenic (Tg) mice exhibited a significant 13% decrease in rCBF during ON vs. OFF periods. During ON periods, an even greater reduction in rCBF for EMF-treated Tg mice was evident when compared to Tg controls. *p<0.05 vs. OFF period by paired t-test. (Fig. 5B) Evaluation of rCBF results from Study I irrespective of genotype revealed that EMF-treated mice had significantly reduced rCBF during both ON and OFF periods. ^★^p<0.05 vs. No EMF; ^★★^p<0.0001 vs. No EMF. (Fig. 5C) At 12 days into EMF treatment for Study II's APPsw+PS1 (Tg) mice, a near-significant rCBF reduction of 19% was present in EMF-treated Tg mice during ON vs. OFF periods. ^†^p = 0.10 for ON vs. OFF (paired t-test); ^#^p<0.05 vs. Tg control during OFF period.

Laser Doppler measurements of regional cerebral blood flow (rCBF) in cerebral cortex were performed at 2 months into EMF treatment for Study I and at 12 days into EMF treatment for Study II. In Study I, control NT and Tg mice (not being given EMF treatment) had very consistent rCBF readings between their sham ON and OFF periods (Fig. 5A). Although NT+EMF mice exhibited no change in rCBF between ON and OFF periods, Tg mice showed a significant 13% decrease in rCBF during the ON period vs. OFF period (Fig. 5A). The decreased rCBF present in Tg mice during the ON period was even greater (↓25%) in relation to rCBF in control Tg mice during their sham ON period. Visual inspect of the data in Fig. 5A revealed rCBF measurements during both OFF and ON periods to be lower in EMF-treated mice compared to control (sham-treated) mice irrespective of genotype. This, in addition to no genotypic differences in rCBF being present for EMF-treated or control mice, warranted combination of individual animal data from both genotypes to determine the main effect of EMF during OFF and ON periods (Fig. 5B). A significant decrease in rCBF was present not only during ON periods for EMF vs. control mice, but also present during OFF periods as well. Thus, EMF effects on rCBF were present not only during ON periods, but also during OFF periods, at 2 months into EMF treatment.

rCBF measurements in Study II only involved Tg mice and at a shorter 12-days into the same daily EMF exposure. As shown in Fig. 5C, control Tg mice had stable and similar rCBF measurements during OFF and sham ON periods. By contrast, a nearly significant (p = 0.10) reduction in rCBF (↓19%) was present in EMF-treated Tg mice during their ON period vs. OFF period at 12 days into EMF exposure. Supportive that a true EMF-induced decrease in rCBF had indeed occurred, 4 out of five Tg+EMF mice had decreases of 7–46% in rCBF during the ON period compared to the OFF period. The significantly higher rCBF present in EMF-treated mice vs. control Tg mice during the OFF period was due to several EMF-treated mice with high rCBF readings during both OFF and ON periods.

### Aβ immunohistochemistry

**Figure 6:**
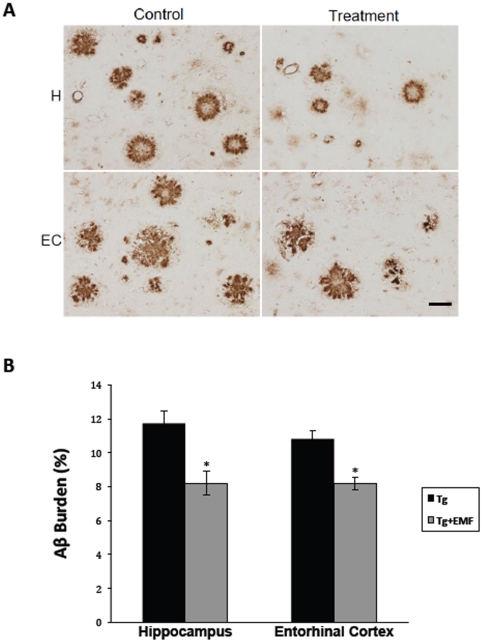
Brain Aβ deposition in APPsw transgenic (Tg) mice at 2 months after EMF treatment (Study I). (Fig. 6A) Photomicrographs showing the visually evident decrease in Aβ deposition in both hippocampus (H) and entorhinal cortex (EC) of EMF-treated mice compared to control/sham mice. Micrometer bar = 50 µm. (Fig. 6B) Quantification of percent Aβ burdens from EMF-treated and control/sham Tg mice. Highly significant reductions in Aβ deposition/aggregation were present in both hippocampus (↓30%) and entorhinal cortex (↓24%) of EMF-treated mice. *p<0.005 vs. Tg controls for that brain area.

After two months of EMF treatment, the very old (23–28 months old) APPsw and NT mice in Study I were euthanized and their brains processed for quantitative analysis of Aβ deposition. As expected, NT mice exhibited no human Aβ immunostaining in their brains irrespective of treatment. Very old Tg controls (Tg), however, had extremely high levels of Aβ deposition in both their hippocampus and entorhinal cortex, bearing Aβ burdens of 11–12% in these two brain areas (Fig. 6B). In sharp contrast, Tg mice that had received two months of EMF treatment exhibited substantial decreases in Aβ burden within both hippocampus (↓30%) and entorhinal cortex (↓24%) compared to Tg controls (Fig. 6B). Thus, EMF treatment reversed pre-existing Aβ deposition/plaque formation. Fig. 6A shows representative photomicrographs of typical Aβ immunostained-plaques from 23–28 months old Tg and Tg+EMF mice, underscoring the substantial reduction in A*β* deposition present in brains of very old Tg mice given a two-month period of daily EMF treatment. Analysis of plasma samples taken at euthanasia revealed no effects of EMF treatment on plasma Aβ1–40 (4620±442 pg/ml for Tg vs. 4885±920 pg/ml for Tg+EMF; p = 0.78) or Aβ1–42 (1452±120 pg/ml for Tg vs. 1175±251 pg/ml; p = 0.30).

## Discussion

We have previously reported that long-term (>6 months) EMF exposure at cell phone level frequencies and SAR levels can protect against or reverse cognitive impairment in Alzheimer's Tg mice, while even having cognitive benefit to normal mice [4]. Moreover, we previously provided the first mechanistic insight into long-term EMF treatment by reporting the ability of such treatment to suppress brain Aβ aggregation/deposition in AD mice, while enhancing brain mitochondrial function and neuronal activity in both Tg and normal mice [4], [5], [20]. The present study extends the above works by administering long-term (2 months) of daily EMF treatment to very old Alzheimer's Tg mice and showing that such treatment can reverse their very advanced brain Aβ aggregation/deposition while providing selected cognitive benefit to both Tg and normal mice. Moreover, these neuropathologic and cognitive benefits occurred without appreciable increases in brain temperature, indicating involvement of non-thermal brain mechanisms (i.e., Aβ anti-aggregation, mitochondrial enhancement, neuronal activity). Finally, the present study is the first to determine the effects of long-term EMF exposure on rCBF, and in the same animals evaluated for cognitive, neuropathologic, and body/brain temperature endpoints. Our finding of an EMF-induced decrease in cortical blood flow raises several interesting mechanisms of action that merit consideration.

### Cognitive and Aβ deposition effects of EMF treatment

Two months of cell phone level EMF treatment (e.g., GSM, 918 MHz, 0.25–1.05 W/kg, pulsed and modulated) improved the cognitive performance of very old (23–27 month old) Tg and NT mice combined in the Y-maze task of spontaneous alternation. This task evaluates general mnemonic function and is not associated with brain Aβ levels/deposition [23]. Thus, generalized mechanisms irrespective of genotype, such as the brain mitochondrial enhancement present by one month into EMF treatment [5], are most likely involved. The present Y-maze results are consistent with our initial study [4] wherein we found Y-maze spontaneous alternation to be significantly increased in NT mice given long-term EMF treatment. By contrast, long-term EMF treatment was not able to reverse the cognitive impairment in two tasks wherein performance is linked to brain Aβ levels/deposition - the circular platform task of spatial/reference memory and RAWM task of working memory [23]. The RAWM task, in particular, is very sensitive to brain Aβ deposition, with poorer working memory performance highly correlated with extent of Aβ deposition in both hippocampus and cortex.

Although the very old Tg mice of this study had extraordinarily high brain Aβ burdens (11–12%) that were substantially reduced (↓24–30%) by EMF treatment, this large quantitative reduction in Aβ deposition was apparently not sufficient for cognitive benefit to become manifest in tasks linked to brain Aβ levels/deposition. A longer EMF treatment period or more effective EMF parameters is probably needed to attain widespread behavioral benefit in these very old Tg mice. In our initial study [4], 6–7 months of daily EMF treatment was required to manifest widespread cognitive benefit in younger Tg mice bearing only around 2% brain Aβ burdens. Parenthetically, animals in the present study were given double the daily EMF exposure (two 2-hour periods) compared to our initial study (two 1-hour periods). For both studies, a more effective removal of Aβ from the brain through greater EMF-induced Aβ disaggregation and ensuing greater removal of resultant soluble Aβ from the brain into the blood would appear to be key to realization of earlier cognitive benefits.

It is important to underscore that an absolute reduction in brain “soluble" Aβ is not required to get EMF-induced cognitive benefits, as we have repeatedly demonstrated for various AD therapeutics including EMF treatment [4], [24], [25]. This is because the disaggregating action of EMF treatment on brain Aβ (from insoluble to soluble forms) appears to shift most soluble Aβ from the cognitive-impairing “oligomeric" form to smaller (innocuous) dimeric/monomeric forms. That is the probable reason why we observed brain mitochondrial enhancement in aged Tg mice given long-term (1 month) EMF treatment despite those treated mice having 5–10× higher soluble Aβ in their brain mitochondria (i.e., most of this soluble Aβ was in innocuous monomeric/dimeric forms) [5]. Such enhanced levels of monomeric/soluble Aβ are also consistent with the lack of EMF-induced reductions in plasma Aβ levels observed in the present study, as well as in our earlier EMF study [4].

Prior to our recent study showing cognitive efficacy of “cell phone-level" EMF exposure administered daily for >6 months to Tg and normal mice [4], animal studies investigating cognitive effects of cell phone level EMF exposure involved “normal" mice/rats receiving daily “head-only" [26]–[28] or “full body" [29] EMF exposure for a relatively short 4–14 days. No cognitive benefits were reported in those studies, nor did longer 2- or 6-month periods of daily head-only EMF exposure impact cognitive performance in normal rats [28]. However, a 5-week period of cell phone level EMF exposure to immature (3 weeks old) rats did improve their rate of learning in the Morris water maze task [30]. It is important to note that future rodent studies emphasize “head-only" EMF exposure over many months and utilize a comprehensive array of cognitive measures/tasks (not simply a single measure/task).

In humans, all cell phone level EMF studies investigating cognitive function have been unilateral and involved either single EMF exposure [15], [16] or daily EMF exposure for 6–27 days [13], [14], with no cognitive effects being reported in either case. However, one study did report that heavy cell phone users evaluated over a 2-year period performed better in a word interference test [2]. Clearly, there is a critical need for long-term, well-controlled EMF studies in humans to evaluate cognitive effects in both normal and cognitive-impaired individuals.

### Body/brain temperature and cerebral blood flow effects of EMF treatment

Before our own recent work [4], [5] and the present study, only one prior animal study investigated the effects of EMF exposure on body/brain temperature and/or cerebral blood flow [31]. That study, involving a single head-only GSM exposure for 18 minutes to anesthetized rats, was at very high frequency (2000 MHz) and very high SAR levels (10–263 W/kg). This acute EMF exposure increased brain temperature in a dose-dependent fashion (by 1–12°C), and increased cortical cerebral blood flow (by 30–70%). In humans, no studies investigating EMF effects on brain temperature have apparently been done in living individuals, and EMF effects on cerebral blood flow have only involved a single, unilateral EMF exposure, with inconsistent results [16]. Thus, for both animals and humans, there had previously been no investigations into long-term EMF effects on brain temperature or cerebral blood flow.

Regarding temperature, our recent studies [4], [5] have investigated both acute and long-term body/brain temperature effects of EMF treatment (i.e., GSM, pulse/modulated at 918 MHz and 0.25–1.05 W/kg), with the following findings: 1) a single day of EMF treatment has no effect on body or brain temperature of either AD Tg or normal mice during ON periods; 2) At 8–9 months into daily EMF treatment, body temperature of both Tg and NT mice is elevated by approximately 1°C during ON periods; and 3) At 1 month into daily EMF treatment, body temperature of aged Tg and NT mice is elevated by around 1°C during ON periods while brain temperatures are either stable (NT mice) or decreased (Tg mice) during ON periods. For both long-term EMF studies in 2) and 3), body temperature always returned back down to normal levels during OFF periods.

The present work extends our aforementioned initial findings by performing two separate temperature-monitoring studies in order to evaluated sub-chronic (12 days) and long-term (6 weeks) effects of daily EMF treatment on both body and brain temperature measurements in very old AD mice and normal mice. During multiple temperature measurements taken over a 6-week period in very old mice that had been behaviorally tested, small (but significant) increases of around 0.5°C in body temperature were evident in both Tg and normal mice. This small increase of <1°C in body temperature during ON periods of long-term EMF treatment is very consistent with that seen in our prior studies [4], [5]. Despite these small, but significant increases in body temperature during ON periods, brain temperature for Tg and normal mice remained stable or was only elevated 0.3–0.4°C through 6 weeks of exposure - far below what would be needed to incur brain/physiologic damage [32]. Thus, the EMF-induced cognitive benefits in mice that we have reported both in our prior report [4] and presently are apparently due to non-thermal brain mechanisms - several of which we have already identified (see last section).

In the sub-chronic (12-day) EMF treatment study, very old APPsw+PS1 (Tg) mice exhibited no change in body or brain temperature during ON periods at both 5 days and 12 days into EMF treatment. This is somewhat in contrast to the long-term study, wherein a significant increase in body temperature during ON periods was already present at 1 week into EMF treatment, although no change in brain temperature occurred (same as in sub-chronic study). The only difference between the two studies, other than temperature recording points, was that double Tg (APPsw+PS1) mice were used in the sub-chronic study, which would have even greater brain Aβ burdens than the APPsw mice used in the long-term study.

At 2 months into daily EMF treatment in the long-term study, Tg mice (but not normal mice) exhibited a significant 13% decrease in rCBF during ON vs. OFF periods. This EMF-induced reduction in rCBF was even greater (↓25%) compared to control Tg mice during sham ON periods. The difference between Tg and NT mice is brain production and aggregation/deposition of Aβ in Tg mice. Earlier studies have provided evidence that EMF treatment increases neuronal activity [16], [19], [21], [33], [34]. As mentioned previously, our very recent findings show that long-term EMF treatment does indeed increase neuronal activity in Tg and NT mice, irrespective of genotype [20]. Since intraneuronal Aβ is synaptically released in greater amounts during increased neuronal activity [35], there is presumably greater efflux of this soluble/monomeric Aβ out of the brain and into the blood during EMF exposure. Inasmuch as vascular Aβ is a well-known constrictor of smooth muscle in resistance vessels (e.g., arterioles), we believe that this enhanced presence of cerebrovascular Aβ due to EMF exposure induces cerebral vasoconstriction and the resulting decreases in rCBF that were observed in Tg mice.

Also in the long-term (2 months) study, rCBF was reduced even during OFF periods in both Tg and normal mice being given EMF treatment. Indeed, when both genotypes were combined to investigate main effects of EMF treatment, rCBF was significantly decreased during both ON (↓23%) and OFF (↓16%) periods. Clearly, some non-specific EMF mechanism is reducing rCBF during OFF periods in both Tg and NT mice. For example, this may be a continuing auto-regulatory response to limit brain heating due to the slight body hyperthermia present during ON periods. Along this line, body hyperthermia (such as that induced by exercise) has been shown to decrease cerebral blood flow in humans by 18% [36], [37]. The reductions in rCBF presently observed during both ON and OFF periods of long-term EMF treatment in Tg and NT mice are consistent with several human PET studies reporting that rCBF is reduced during single exposure EMF treatment [18], [38].

Similar to rCBF results from the long-term EMF study, evaluation of rCBF at 12 days into EMF treatment for APPsw+PS1 (Tg) mice in the sub-chronic study revealed a near significant 19% decrease in rCBF during ON periods. Indeed, 4 of 5 Tg-treated mice exhibited rCBF decreases of 7–46%. Since there was no increase in body temperature during ON periods, there was no need for themoregulatory mechanisms to limit CBF to the brain. However, it is likely that during ON periods, elevated vascular Aβ caused a modest vasoconstriction in the brain and the ensuing decrease in CBF that was observed.

### Mechanisms of long-term EMF action and evidence for EMF safety

**Figure 7:**
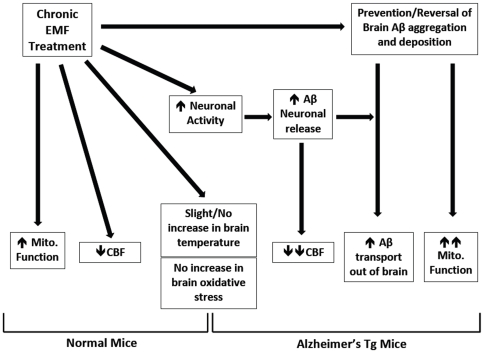
Summary diagram depicting both confirmed and proposed mechanisms of long-term EMF action in normal mice and Alzheimer's transgenic (Tg) mice. Long-term EMF actions that we have confirmed include prevention/reversal of brain Aβ aggregation, brain mitochondrial enhancement, and reduced cortical cerebral blood flow (CBF). These long-term EMF actions occur through slight/no increase in brain temperature and without increasing brain oxidative stress/damage.

Results from the present study, in concert with those from our prior three studies [4], [5], [20], are beginning to provide critical mechanistic insight into the ability of long-term, high frequency EMF exposure to benefit cognitive function in normal and AD mice. Fig. 7 summarizes our current understanding of those mechanisms, which are relevant to human long-term EMF exposure as well. Although this summary diagram is the result of long-term studies involving GMS-modulated and pulsed EMF treatment at specific parameters (918 MHz, 0.25–1.05 W/kg), different combinations of frequency/SAR levels will likely provide more robust mechanistic actions within this circuit and expand it, resulting in greater or more rapid cognitive benefit.

As depicted in Fig. 7 for AD mice, high frequency EMF treatment would appear to exert two complementary actions that ultimately result in enhanced Aβ removal/efflux from the brain: 1) prevention and reversal of brain Aβ aggregation/deposition [4], and 2) increased neuronal/EEG activity [16], [20], [19]–[21], [33], [34]. EMF treatment's suppression of extracellular and intracellular Aβ aggregation, combined with enhanced synaptic release of intra-neuronal Aβ during increased neuronal activity [35], result in soluble monomergic forms of free Aβ in the brain parenchyma - Aβ forms that can be readily transported across the blood-brain barrier [39] and into the blood for eventual degradation. As previously mentioned, soluble/monomeric Aβ is a powerful vasoconstrictor [40], [41], which is probably key to the substantial decrease in rCBF present during EMF ON periods in Tg mice. Since Aβ is not a factor for EMF effects in normal mice, normal mice incur a less robust, generalized decrease in CBF through some as yet unidentified mechanism (e.g., compensatory to EMF-induced increases in body temperature). Similarly, long-term EMF treatment to Tg mice induces large enhancements in brain mitochondrial function due to disaggregation of mitochondrial-impairing oligomeric Aβ in neurons, with a lesser enhancement present in normal mice due to an as yet unidentified mechanism [5].

All of the aforementioned EMF mechanisms occur in mice with only a slight (or no) increase in brain temperature [5] and no increase in brain oxidative stress/damage [4]. Indeed, examination of both peripheral and brain tissues from animals given daily EMF treatment for over 8 months has revealed no tissue abnormalities [4], including no increase in DNA damage to blood cells from these same animals [Cao et al., unpublished observations]. The lack of deleterious brain and peripheral effects in such long-term EMF studies, in combination with recent epidemiologic human studies also reporting no consistent evidence for EMF-induced health problems [10]–[12], underscores the mounting evidence that high frequency EMF treatment over long periods of time, could be a safe and novel disease-modifying therapeutic against AD.

## Materials and Methods

### Ethics statement

All animal procedures were performed in AAALAC-certified facilities under protocol #R3258, approved by the University of South Florida Institutional Animal Care and Use Committee.

### Animals

For both studies of this work, a total of 41 aged mice derived from the Florida Alzheimer's Disease Research Center's colony were included. Each mouse had a mixed background of 56.25% C57, 12.5% B6, 18.75% SJL, and 12.5% Swiss-Webster. All mice were derived from a cross between heterozygous mice carrying the mutant APPK670N, M671L gene (APPsw) with heterozygous PS1 (Tg line 6.2) mice, which provided offspring consisting of APPsw+PS1, APPsw, PS1, and NT genotypes. After weaning and genotyping of these F10 and F11 generation offspring, APPsw and NT mice were selected for a long-term behavioral study (Study I), while APPsw+PS1 mice were selected for a follow-up, shorter duration temperature/cerebral blood flow-monitoring study (Study II) - aged APPsw were not available for the ensuing Study II. All mice were housed individually after genotyping, maintained on a 12-hour dark and 12-hour light cycle with *ad libitum* access to rodent chow and water.

### Study I: Two-month EMF Treatment Study

At 21–26 months of age, APPsw Tg mice (n = 17) and NT littermates (n = 10) were first evaluated in RAWM task of working memory (see Behavioral testing protocols) to establish baseline cognitive performance for both genotypes prior to EMF treatment. Based on pretreatment performance in the RAWM task, Tg and NT groups were each divided into two performance-balanced sub-groups as follows: Tg controls (n = 8), Tg+EMF (n = 9), NT controls (n = 5), and NT+EMF (n = 5). Tg and NT mice to be exposed to EMFs had their cages placed within a large Faraday cage, which contained an EMF generator antenna that provided two 2-hour periods of EMF treatment per day (see EMF treatment protocol). At 22–27 months of age (one month into EMF treatment), all mice were started on a one-month series of behavioral tasks. EMF treatment was continued during the one-month behavioral testing period, with all testing performed during “OFF" periods in between the two daily EMF treatments. Body and brain temperature measurements were performed just prior to initiation of EMF treatment and at 1, 3, and 6 weeks into EMF treatment (see Body/brain temperature determinations). Doppler recordings of rCBF were taken at 2 months in EMF treatment (see rCBF determinations). On the day following rCBF measurements, animals were euthanized at 23–28 months of age, during which a blood sample was taken and brains were perfused with isotonic phosphate-buffered saline (PBS). The caudal brain was then paraffin-embedded and processed for A*β* immunohistochemical staining, while the remaining forebrain was sagitally bisected and dissected into hippocampus and cortical areas that were quick-frozen for neurochemical analyses. Plasma was analyzed for both Aβ1–40 and Aβ1–42.

### Study II: 12-day EMF Treatment Study

At 22 months of age, 11 APPsw+PS1 Tg mice were divided into two groups of 5–6 mice each. One group was placed into the faraday cage for two daily EMF exposures exactly as for mice in the 2-month EMF Treatment Study (see EMF treatment protocol). The other group served as EMF controls, housed in a completely separate room with an identical environment without EMF treatment. Body and brain temperature recordings were taken from all mice just prior to onset of the first EMF treatment, as well as on the 5^th^ day and 12^th^ day into EMF treatment. Concurrent with temperature recording on Day 12, cerebral blood flow measurements were also taken.

### EMF treatment protocol

Tg and NT mice given EMF treatment were individually housed in cages within a large Faraday cage, which also housed the antenna of an EMF generator providing two 2-hour periods of electromagnetic waves per day (early morning and late afternoon). Each EMF exposure was at 918 MHz frequency, involved modulation with Gaussian minimal-shift keying (GMSK) signal, and was pulsed/non-continuous with carrier bursts repeated every 4.6 ms, giving a pulse repetition rate of 217 Hz. The electrical field strength varied between 17 and 35 V/m. This resulted in calculated SAR levels that varied between 0.25 and 1.05 W/kg. Calculated SAR values have been shown to correspond closely with measured SAR values [42]. SAR was calculated from the below equation, with σ (0.88 sec/m) and ρ (1030 kg/m^3^) values attained from Nightingale et al. [43]:
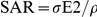



σ = mean electrical conductivity of mouse brain tissue.

ρ = mass density of mouse brain.

E = electrical field strength.

For the 2-month and 12-day periods of EMF treatment given to mice in Study's I and II, respectively, cages of individually-housed mice were maintained within the Faraday cage (1.2×1.2×1.2 m^3^) and arranged in a circular pattern. Each cage was approximately 26 cm from a centrally located EMF-emitting antenna. The antenna was connected to a Hewlett–Packard ESG D4000A digital signal generator (Houston, TX, USA) set to automatically provide two 2-hour exposures per day. With a 12-hour light ON/OFF cycle, the 2-hour daily exposures occurred in early morning and late afternoon of the lights on period. Sham-treated control Tg and NT mice were located in a completely separate room, with identical room temperature as in the EMF exposure room and with animals individually housed in cages that were arranged in the same circular pattern.

### Behavioral Testing Protocols

Prior to EMF treatment, all mice in Study I were behaviorally tested for 10 days in RAWM task of working memory to determine baseline cognitive performance in this task. Daily EMF treatment was then started, with behavioral testing initiated at one month into EMF treatment and occurring between early morning and late afternoon EMF treatments. One-day tasks of sensorimotor function were initially carried out (open field activity, balance beam, string agility), followed by a one-day Y-maze task (locomotor activity, spontaneous alternation), then RAWM Test I (4 days), circular platform performance (4 days), RAWM Test II (4 days), then finally the visual cliff test of visual acuity (1 day). Although the methodologies for all of these tasks have been previous described and are well established [44]–[46], a brief description of each task is provided below:

#### Open field activity

Open field activity was used to measure exploratory behavior and general activity. Mice were individually placed into an open black box 81×81 cm with 28.5-cm high walls. This area was divided by white lines into 16 squares measuring 20×20 cm. Lines crossed by each mouse over a 5-minute period were counted.

#### Balance beam

Balance beam was used to measure balance and general motor function. The mice were placed on a 1.1-cm wide beam, suspended above a padded surface by two identical columns. Attached at each end of the beam was an escape platform. Mice were placed on the beam in a perpendicular orientation and were monitored for a maximum of 60 secs. The time spent by each mouse on the beam before falling or reaching one of the platforms was recorded for each of three successive trials. If a mouse reached one of the escape platforms, a time of 60 secs was assigned for that trial. The average of all three trials was utilized.

#### String agility

String agility was used to assess forepaw grip capacity and agility. Mice were placed in the center of a taut cotton string suspended above a padded surface between the same two columns as in the balance beam task. Mice were allowed to grip the string with only their forepaws and then released for a maximum of 60 secs. A rating system, ranging between 0 and 5, was employed to assess string agility for a single 60-sec trial.

#### Y-maze spontaneous alternation

Y-maze spontaneous alternation was used to measure general activity and basic mnemonic function. Mice were allowed 5 minute to explore a black Y-maze with three arms. The number and sequence of arm choices were recorded. General activity was measured as the total number of arm entries, while basic mnemonic function was measured as a percentage of spontaneous alternation (the ratio of arm choices different from the previous two choices divided by the total number of entries).

#### Circular platform

Circular platform was used to measure spatial/reference learning and memory. Mice were placed on a 69-cm circular platform with 16 equally spaced holes on the periphery of the platform. Underneath only one of the 16 holes was a box filled with bedding to allow the mouse to escape from aversive stimuli (e.g. two 150-W flood lamps hung 76 cm above the platform and one high-speed fan 15 cm above the platform). Each mouse was administered one 5-minute trial per day to explore the area. For the single trial administered on each of four test days, mice were placed in the center of the platform facing away from their escape hole (which differed for each mouse). Escape latency was measured (maximum of 300 secs) each day. Data was statistically analyzed in two 2-day blocks.

#### RAWA

RAWA task of spatial working memory involved use of an aluminum insert, placed into a 100 cm circular pool to create 6 radially distributed swim arms emanating from a central circular swim area. An assortment of 2-D and 3-D visual cues surrounded the pool. The latency and number of errors prior to locating which one of the 6 swim arms contained a submerged escape platform (9 cm diameter) was determined for 5 trials/day over 10 days of pre-treatment testing. There was a 30-minute time delay between the 4^th^ trial and the 5^th^ trial (T5; memory retention trial). The platform location was changed daily to a different arm, with different start arms for each of the 5 trials semi-randomly selected from the remaining 5 swim arms. During each trial (60-sec maximum), the mouse was returned to that trial's start arm upon swimming into an incorrect arm and the number of seconds required to locate the submerged platform was recorded. If the mouse did not find the platform within a 60-sec trial, it was guided to the platform for the 30-sec stay. The latency and number of errors during Trial 1 (T1) are chance performance since the animal does not know where the submerged platform is for the first trial of any given day. Latency and errors during the last trial (Trial 5; T5) of any given day are considered indices of working memory and are temporally similar to the standard registration/recall testing of specific items used clinically in evaluating AD patients. Data for T1 and T5 were statistically analyzed in two-day blocks, as well as overall, for the 10-day of pretreatment RAWM testing, the 4-day of RAWM Test I, and the 4-day of RAWM Test II. Because the final block of testing is most representative of true working memory potential in this task, results from the last 2-day block of testing are presented for all three RAWM test periods.

#### Visual Cliff

Visual Cliff was utilized on the last day of behavioral testing to evaluate vision/depth perception. A wooden box has two horizontal surfaces, both of which have the same bold pattern, but one surface of which is 10–12 inches below the other. A sheet of clear Plexiglass is placed across the entire horizontal surface, providing the visual appearance of a cliff. An animal with poor vision/depth perception cannot detect the “cliff" and will move without hesitation across the cliff, resulting in a score of “1". An animal with good vision will pause/hesitate at the cliff before crossing it and is scored a “2".

### Body/brain temperature determinations

For body/brain temperature determinations of mice in both Studies I and II, body temperature was taken via rectal probe and brain temperature via temporalis muscle probe. Prior studies have demonstrated that temporalis muscle temperature very accurately reflects brain temperature in rodents [47], [48]. Temperature determinations during EMF treatment (ON periods) were taken near the end of the morning EMF treatment, while temperature determinations during OFF periods were in early afternoon (mid-way between the two daily EMF treatments). Each measurement only took a couple of minutes for each mouse.

### rCBF determinations

In cerebral cortex, rCBF measurements during the ON period were taken near the end of either the morning EMF treatment session (Study I) or the afternoon treatment session (Study II). rCBF measurements during the OFF period were taken in early afternoon, mid-way between both EMF treatment sessions. For each measurement, anesthetized (equithesin 300 mg/kg i.p.) animals underwent rCBF measurement using laser Doppler flowmetry (PF-5010, Periflux system, Järfälla, Sweden) with relative flow values expressed as perfusion units [49], [50]. All rCBF measurements were conducted with the animal fixed in a Kopf stereotaxic apparatus, with the probe placed at the level of the dura directly above a small skull opening. Using a micromanipulator, two probes (probe 411, 0.45 mm in diameter) were positioned to cortical coordinates of 1.3 mm posterior to the bregma and 2.8 mm to each side of midline on the intact skull, being careful to avoid pial vessels after reflection of the skin overlying the calvarium. Because mouse skull and subarachnoid space are very thin, transcranial measurements of rCBF are consistent with craniectomy measurements [51]. The rCBF of both hemispheres were continuously measured for 15 minutes and averaged for each determination. All rCBF data was continuously stored in a computer and analyzed using the Perimed data acquisition and analysis system.

### Aβ immunohistochemistry and image analysis

At the level of the posterior hippocampus (bregma −2.92 mm to −3.64 mm), five 5 µm sections (150 µm apart) were taken from each mouse brain using a sliding microtome (REM-710, Yamato Kohki Industrial, Asaka, Saitama, Japan). Immunohistochemical staining was performed following the manufacturer's protocol using aVectastainABC *Elite* kit (Vector Laboratories, Burlingame, CA) coupled with the diaminobenzidine reaction, except that the biothinylated secondary antibody step was omitted. Used as the primary antibody was a biothinylated human Aβ monoclonal antibody (clone 4G8; 1∶200, Covance Research Products, Emeryville, CA). Brain sections were treated with 70% formic acid prior to the pre-blocking step. 0.1 M PBS (pH 7.4) or normal mouse serum (isotype control) was used instead of primary antibody or ABC reagent as a negative control. Quantitative image analysis was done based on previously validated method [52]. Images were acquired using an Olympus BX60 microscope with an attached digital camera system (DP-70, Olympus, Tokyo, Japan), and the digital image was routed into a Windows PC for quantitative analysis using SimplePCI software (Hamamatsu Photonics, Hamamatsu, Shizuoka, Japan). Images of five 5-µm sections (150 µm apart) through both anatomic regions of interest (hippocampus and entorhinal cortex) were captured from each animal, and a threshold optical density was obtained that discriminated staining from background. Each region of interest was manually edited to eliminate artifacts, with Aβ burden data reported as percentage of immune-labeled area captured (positive pixels) relative to the full area captured (total pixels). Each analysis was done by a single examiner blinded to sample identities.

### Plasma Aβ levels

Aβ1–40 and 1–42 levels were determined from plasma samples by using ELISA kits (KHB3482 for 40, KHB3442 for 42, Invitrogen, CA). Standard and samples were mixed with detection antibody and loaded on the antibody pre-coated plate as the designated wells. HRP-conjugated antibody was added after wash, and substrates were added for colorimetric reaction, which was then stopped with sulfuric acid. Optical density was obtained and concentrations were calculated according a standard curve.

### Statistical Analysis

Data analysis of physiologic and neurohistologic measurements, as well as all one-day behavioral measures, were performed using ANOVA followed by Fisher's LSD *post hoc* test. For the multiple-day behavioral tasks (RAWM and circular platform), initial ANOVA analysis of 2-day blocks and overall were followed by analysis of *post hoc* pair-by-pair differences between groups via the Fisher LSD test. For temperature and blood flow measurements within the same animal, paired *t*-tests were employed. All data are presented as mean ± SEM, with significant group differences being designated by p<0.05 or higher level of significance.
